# Real-World Laboratory Analysis of Molecular Biomarkers in Multiple Sclerosis Centers in Central-Eastern European Countries Covering 107 Million Inhabitants

**DOI:** 10.3390/ijms26178274

**Published:** 2025-08-26

**Authors:** Anett Járdánházy, Thomas Berger, Harald Hegen, Bernhard Hemmer, Halina Bartosik-Psujek, Vanja Basic Kes, Achim Berthele, Jelena Drulovic, Mario Habek, Dana Horakova, Alenka Horvat Ledinek, Eva Kubala Havrdova, Melinda Magyari, Konrad Rejdak, Cristina Tiu, Peter Turcani, Krisztina Bencsik, Zsigmond Tamás Kincses, László Vécsei

**Affiliations:** 1 Department of Neurology, Albert Szent-Györgyi Faculty of Medicine, Albert Szent-Györgyi Clinical Centre, University of Szeged, Semmelweis Street 6, 6725 Szeged, Hungary; anettjardanhazy@gmail.com (A.J.);; 2 Department of Neurology, Medical University of Vienna, Waehringer Guertel 18–20, 1090 Vienna, Austria; 3 Comprehensive Center for Clinical Neurosciences & Mental Health, Medical University of Vienna, Währinger Gürtel 18–20, 1090 Vienna, Austria; 4 Department of Neurology, Medical University of Innsbruck, Christoph-Probst-Platz 1, Innrain 52, 6020 Innsbruck, Austria; 5 Department of Neurology, School of Medicine and Health, Technical University Munich, Arcisstraße 21, 80333 Munich, Germany; 6 Munich Cluster for System Neurology (SyNergy), Feodor-Lynen-Str. 17, 81377 Munich, Germany; 7 Department of Neurology, Institute of Medical Sciences, Medical College of Rzeszow University, 1A Warzywna Street, 35-310 Rzeszow, Poland; 8 Department of Neurology, University Hospital Sestre Milosrdnice, Vinogradska Cesta 29, 10000 Zagreb, Croatia; 9 Clinic of Neurology, University Clinical Centre of Serbia, Dr Subotica 6, 11000 Belgrade, Serbia; 10 Department of Neurology, University Hospital Center Zagreb, School of Medicine, University of Zagreb, Kišpatićeva Ulica 12, 10000 Zagreb, Croatia; 11 Department of Neurology and Center of Clinical Neuroscience, First Faculty of Medicine, Charles University, General University Hospital, Kateřinská 30, Praha 2, 128 21 Prague, Czech Republic; 12 Department of Neurology, University Clinical Centre Ljubljana, Zaloška Cesta 2a, 1000 Ljubljana, Slovenia; 13 Danish Multiple Sclerosis Center, Copenhagen University Hospital, Rigshospitalet, Blegdamsvej 9 DK, 2100 Copenhagen, Denmark; 14 Department of Neurology, Medical University of Lublin, ul. Jaczewskiego 8, 20-954 Lublin, Poland; 15 Department of Neurology, University Hospital Bucharest, Splaiul Independentei Nr. 169, Sector 5, 050098 Bucharest, Romania; 16 1st Department of Neurology, Faculty of Medicine, Comenius University, University Hospital Bratislava, Mickiewiczova 13, 813 69 Bratislava, Slovakia; 17 Department of Radiology, Albert Szent-Györgyi Faculty of Medicine, Albert Szent-Györgyi Clinical Centre, University of Szeged, Semmelweis Street 6, 6725 Szeged, Hungary; 18 MTA-SZTE Neuroscience Research Group, Semmelweis Street 6, 6725 Szeged, Hungary

**Keywords:** molecular biomarkers, Multiple Sclerosis (MS), cerebrospinal fluid (CSF), oligoclonal bands (OCB), Intrathecal IgG synthesis, kappa free light chain (κFLC), neurofilament light chain (NfL), anti-drug antibodies (ADA), multicentric survey, Central-Eastern European countries

## Abstract

A multicenter molecular biomarker survey was conducted in Multiple Sclerosis (MS) centers across Central-Eastern European countries, encompassing a population of 107 million. Our aim was to provide a “snapshot” for future studies investigating the use of molecular biomarkers in MS. A self-report questionnaire was distributed via email to MS centers in seven Central-Eastern European countries (Croatia, Czech Republic, Poland, Romania, Serbia, Slovakia, and Slovenia) and to four reference centers (two in Austria, one in Germany, and one in Denmark), focusing on cerebrospinal fluid (CSF) analysis and molecular biomarkers in MS. Responding centers routinely request CSF oligoclonal band (OCB) testing in suspected MS cases, although no consensus exists on the number of CSF-restricted bands required to define OCB positivity, either within or between countries. More than half of the surveyed centers in the Czech Republic, Slovakia, Slovenia, and the reference centers request kappa free light chain (κFLC) testing in patients with suspected MS. Neurofilament light chain (NfL) is frequently used as a molecular biomarker for MS in Romania, Slovakia, and the reference centers. In summary, besides the use of CSF-specific OCB there is no consensus among the surveyed countries regarding the use of molecular biomarkers in MS.

## 1. Introduction

Multiple Sclerosis (MS) is a chronic, inflammatory, immune-mediated, demyelinating, and neurodegenerative disease of the central nervous system (CNS) [1,2]. It can cause various symptoms (e.g., disturbances in movement, coordination, bulbar and visual functions; sensory deficits; cognitive impairment; fatigue and psychiatric conditions) depending on the localization of the lesions. The diagnosis of MS is based on physical examinations, neuroimaging, and blood and cerebrospinal fluid (CSF) analysis [1,3]. The McDonald criteria, first published in 2001, were most recently updated in 2017 [4]. In 2024 Xavier Montalban presented a new revised proposal at the ECTRIMS Congress in Copenhagen. In its 2017 version the presence of IgG oligoclonal bands (OCB) in CSF can substitute the criterion dissemination in time. Thus, this enables early MS diagnosis in patients experiencing a first CNS-demyelinating event (clinically isolated syndrome, CIS) who meet the criteria for dissemination in space [1,4]. In the planned new version (2024) the kappa free light chain (κFLC) index is interchangeable with OCB. Using highly effective disease-modifying therapies (DMTs) as early as possible provides an opportunity to delay long-term progression and maintain a good quality of life [1]. New CSF and serum biomarkers can play an important role in the early diagnosis and in the estimation of prognosis [1,3]. The molecular biomarkers used in clinical practice and potential biomarkers in MS are summarized in Table 1 (based on [1,3,5,6]).

The pathogenesis of MS is not completely known. Myelin and axonal damage are caused by T and B lymphocytes, microglia/macrophages, cytokines, antibodies, and complement [6]. Moreover, both acute and chronic inflammation are major neuropathologic hallmarks. The MS plaques are territories of demyelination associated with inflammation or axonal injury, mainly affecting the white matter. These lesions can be detected by radiological examination (magnetic resonance imaging (MRI)) [6], typically appearing in periventricular, juxtacortical, infratentorial white matter. The interconnections between molecular biomarkers and the pathological processes can be found in the paper of Di Filippo et al. [6].

B cells produce IgG and OCB intrathecally in MS [7]. OCB positivity is usually defined by two or more IgG OCB in the CSF (without their appearance in the serum) [8]. Laboratory testing methods for oligoclonal bands are isoelectric focusing followed by immunofixation [9]. They are with high costs, need a specialist, and interpretation is also subjective [1]. The sensitivity of CSF IgG OCB is 72.2% and the specificity is 95.2% [10]. The presence of IgG OCB in the CSF indicates a higher risk of MS in patients with CIS [8]. The intrathecal IgG synthesis can also be determined by quantitative methods, i.e., measurement of IgG in CSF and serum, followed by calculation of an intrathecal fraction. Different formulae have been suggested including the Auer and Hegen, the Reiber formulae or the IgG index. The IgG index > 0.7 indicates a higher intrathecal B cell response and is associated with MS progression [11]. In 2020, Zheng et al. [12] and Simonsen et al. [13] found the IgG index > 0.7 was well-predictive for OCB positivity, but it could not replace OCB in MS diagnosis [14]. Quantitative methods show a lower diagnostic sensitivity than OCB.

Kappa free light chains are produced by B cells [15,16], and can be piled up in the CSF in case of inflammatory diseases of the CNS [17]. Kappa free light chains (κFLC) can be measured by nephelometry or turbidimetry, which are reliable, cost-effective, and straightforward methods [18]. κFLC concentrations can be measured both in the CSF and serum, followed by calculation of the κFLC index or an intrathecal κFLC fraction (formulas can be found in [18]). Some centers just use the absolute CSF κFLC concentration. κFLC index has been recommended and a recent meta-analysis showed a diagnostic sensitivity of 88% and specificity of 89% which was similar to OCB with 85% sensitivity and 92% specificity [18]. High CSF κFLC levels have been associated with predicting conversion from CIS to MS [19,20]. The limitation of the κFLC index is that there is no consensus about its diagnostic cut-off values [3].

The components of Neurofilament proteins are light, medium, or heavy chains, and among them neurofilament light chain (NfL) is a marker of neurodegeneration. A promising biomarker in MS monitoring and treatment follow-up is the neurofilament light chain. NfL is located in the neuronal cytoskeleton; it enters the interstitial fluid in connection with axon injury and neurodegeneration [21] (but NfL is not solely proof of neurodegeneration, the destruction of axons happens due to inflammation). In case of neuroaxonal injury its level is raised both in the CSF and serum. Electrochemiluminescence and single-molecule array assays are highly sensitive and they were developed to become eligible for serum NfL measurements [22]. A summary about these methods in various MS clinical studies can be found in the review of Kouchaki et al. [23]. Increased level of NfL (in the CSF and blood) can be found not only in MS, but also in other neurodegenerative disorders such as dementias (Alzheimer or frontotemporal type), ALS, brain injury, stroke, etc. [21,24,25,26,27,28,29,30,31,32]. In MS serum NfL is an independent predictive biomarker for CIS conversion to clinically defined MS [33]. Blood NfL level is related to the MS disease activity and it has predictive value [34]. In 2022 Benkert et al. [35] aimed to assemble a reference database with age- and body mass index-corrected sNfL values in MS. The limitation of NfL measurement is that there is no consensus regarding NfL cut-off values in clinical practice [23]. In addition, some studies report the usefulness of NfL in MS treatment follow-up, as nataliumab, ocrelizumab, rituximab, mitoxantron, and fingolimod were found to decrease the NfL levels [34,36,37,38,39,40,41,42,43].

Since molecular biomarkers play a major role in establishing the diagnosis, in monitoring the progression of the disease, in making therapeutic decisions, and in assessing the effectiveness of therapy, strict adherence to international standards is essential, along with continued advancement (e.g., identification of new markers using mass spectrometry and other advanced analytical technologies and their introduction into clinical practice). This study aimed to determine a “snapshot” of the use of molecular biomarkers in MS in different Central-Eastern European Countries, which covers 107 million inhabitants.

## 2. Results

### 2.1. General Information About the Surveyed Centers

First, we assessed the organizational structure of the institutions. The results are summarized in Table 2.

Most of the surveyed centers have neurology in-bed patients and a specialized MS clinic. A research agenda focused on MS and biomarker development was present in ≥50% of the surveyed centers in Slovakia, Slovenia, and the reference centers.

Most of the centers treat *30–100 (numbers are marked with italic)* or more than 100 MS patients per month (Croatia *3/5*, *60% (treat 30–100 MS patients per month)* or 1/5, 20% (treat more than 100 MS patients per month), Czech Republic *1/9*, *11%* or 8/9, 89%, Poland *8/20*, *40%* or 11/20, 55%, Romania *6/14*, *43%* or 6/14, 43%, Serbia *1/5*, *20%* or 4/5, 80%, Slovakia *3/5*, *60%* or 2/5, 40%, Slovenia *1/2*, *50%* or 1/2, 50%, reference centers *0/4*, *0%* or 4/4, 100%).

In all examined centers, the laboratory analysis of the CSF takes place in their institution, except for three centers in Romania (3/14, which is 21.5% of the Romanian participating centers), one in the Czech Republic (1/9, 11%), and one in Poland (1/20, 5%), where it is performed in a private laboratory, or in another hospital. The laboratories in these institutions is operated by Laboratory Medicine in 100% of the responding centers in Croatia (5/5), Romania (11/11), and Slovenia (2/2). In the Czech Republic, Poland, and Serbia, it is mostly operated by Laboratory Medicine, only (1/8) 12.5%, (1/19) 5%, and (1/5) 20% of the centers answered Neurology, one (1/8, 12.5%) center in Czech Republic answered Immunology, and one (1/19, 5%) in Poland the diagnostic unit. In Slovakia the centers marked Neurology (2/5, 40%) and Laboratory Medicine (2/5, 40%) in equal proportion, and one (1/5, 20%) denoted Biochemistry. The situation is quite different in reference centers, because it runs under Neurology in (3/4) 75%. In one reference center (1/4) (25%), it belongs to the Laboratory Medicine and Division of Neuropathology and Neurochemistry, Department of Neurology.

**Figure 1:**
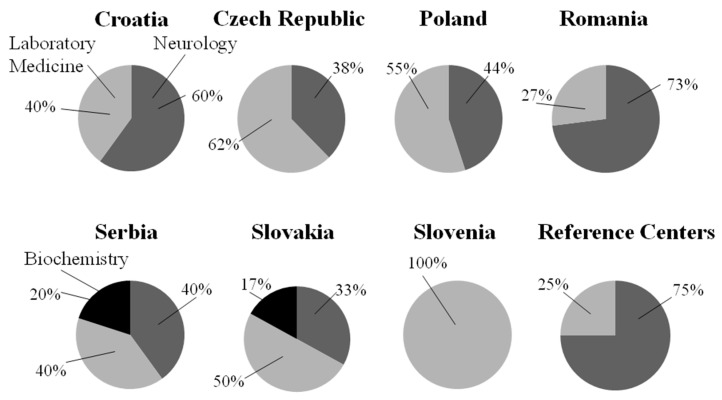
The responsibility for the interpretation of CSF results in different countries. Neurology is marked with dark gray, Biochemistry with black, and Laboratory Medicine with light gray.

The answers to the question—of which discipline is responsible for the interpretation of CSF results (Neurology, Laboratory Medicine, or Biochemistry)—can be seen in Figure 1.

In addition, one center in Czech Republic marked Immunology, and one center in Austria answered Division of Neuropathology and Neurochemistry, Department of Neurology.

### 2.2. Cerebrospinal Fluid Analysis

All surveyed centers in all participating countries always perform lumbar puncture (LP) for CSF analysis in suspected MS cases, except in Romania and Poland. In Romania only (6/14) 43% of the centers always perform the LP, (8/14) 57% of the Romanian centers carry out LP for CSF analysis when magnetic resonance imaging (MRI) is inconclusive, (6/14) 43% when clinical presentation is inconclusive in patients with suspected MS, and (1/14) 7% for research purposes. In Poland the majority of the centers always perform the LP (17/20) 85%, only (2/20) 10% when MRI is inconclusive, and (1/20) 5% when clinical presentation is inconclusive.

100% of the Croatian, Polish, Serbian, Slovakian, Slovenian, and the reference centers answered that both the diagnosis and the differential diagnosis were the main reasons to perform CSF analysis in patients with suspected MS. One Czech center (1/9, 11%) performs it only for diagnostic purposes and one Romanian center (1/14, 7%) only for differential diagnostic purposes; the other centers of these countries also use it for both reasons.

On the question of how long it takes to obtain the report on routine CSF parameters we see rather diverse results even within individual countries. In Croatia it is less than 1 day—40%/*1 day (marked with italic)*—*20%*/more than 1 day—40%; in Czech Republic 78%/*11%*/11%; in Poland 75%/*10%*/15%; in Romania 36%/*14%*/50%; in Serbia 80%/*20%*/0%; in Slovakia 40%/*20%*/40%; in Slovenia 50%/*0%*/50%; and in the reference centers 75%/*25%*/0%.

**Figure 2:**
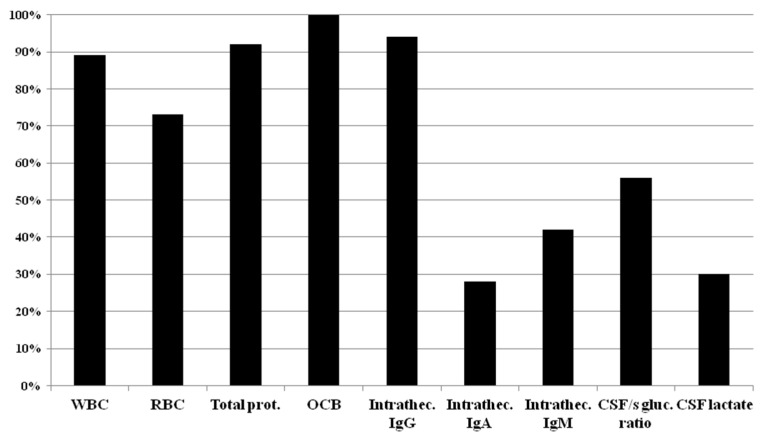
CSF routine parameters usually performed in patients with suspected MS.

Routine CSF parameters usually requested by surveyed centers in suspected MS cases are summarized in Figure 2.

WBC: White blood cell count; RBC: Red blood cell count; Total protein; OCB: Oligoclonal bands; Intrathecal IgG synthesis; Intrathecal IgA synthesis; Intrathecal IgM synthesis; CSF/serum glucose ratio; CSF lactate.

In Table 3 the details of the routine CSF parameters are presented by countries.

As can be seen from the results, majority of the centers use white blood cell count, red blood cell count, total protein, OCB, and intrathecal IgG. To our question of which CSF parameters are considered as MS specific, ≥50% of the responding centers marked the CSF-restricted OCB. Only in Czech Republic the denotation of the intrathecal kappa free light chain was (9/9) 100%, and in the other centers the choice of intrathecal kappa free light chain was ≤50% (Table 4).

On the question of “what is the number of CSF-OCB that is used by your laboratory to define OCB positivity?” we only received a homogeneous answer from the Czech Republic, where (9/9) 100% of the responding centers indicated ≥2 CSF bands. There is no clear consensus in the other countries (see the details in Table 5).

Most of the responding centers from all participated countries also apply the IgG index to calculate the intrathecal fraction of IgG (Croatia: (4/5) 80%; Czech Republic: (6/9) 67%; Poland: (15/20) 75%; Romania: (11/14) 78.5%; Serbia: (4/5) 80%; Slovakia (3/5) 60%; Slovenia (1/2) 50%; reference centers: (3/4) 75%). The use of Reibergram is mostly below 50%, except Slovakia, where it is 100% (5/5).

It takes more than 3 days to obtain the report of OCB in the majority of the centers (Croatia: (4/5) 80%; Czech Republic: (7/9) 78%; Poland: (18/20) 90%; Romania: (13/14) 93%; Serbia: (5/5) 100%; Slovenia: (2/2) 100%; reference centers: (3/4) 75%). In Slovakia, 3 days or more and 3 days are equal (2/5)-(2/5) 40%-40%.

### 2.3. Determination of Kappa Free Light Chain (κFLC) in Patients with Suspected MS

**Figure 3:**
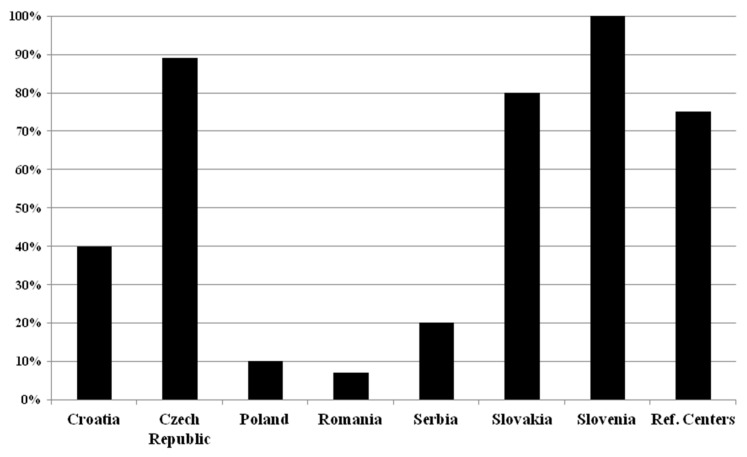
Kappa free light chain (κFLC) determination in patients with suspected MS in different countries.

Our results about the determination of κFLC in patients with suspected MS is summarized in Figure 3.

In Croatia; in Czech Republic; in Poland; in Romania; in Serbia; in Slovakia; in Slovenia; in the reference centers.

In Croatia, only (2/5) 40% of the participated MS centers ask for the determination of κFLC. All of them prefer the κFLC index (2/2) (100%). In Czech Republic the majority of the centers (8/9) (89%) request the κFLC determination; (5/8) 62.5% of these respondents prefer the κFLC index, and the rest the CSF κFLC concentration. In Poland, only two of the centers (2/20) (10%) ask to determine only the κFLC index. In Romania only one investigated center (1/14) (7%) uses CSF κFLC concentration (just in selected cases); the others do not ask for κFLC determination. In Serbia also only one of the surveyed centers (1/5) (20%) asks to determine the κFLC index. In Slovakia (4/5) 80% of the centers seek the determination of κFLC, and in the majority of the cases they ask for the CSF κFLC concentration (3/4) (75%). In Slovenia, (2/2) 100% of the centers ask for the determination of κFLC index and (1/2) 50% for CSF κFLC concentration. In the reference centers κFLC index is only preferred (3/3) (100%).

### 2.4. Other CSF and/or Blood Biomarkers in Patients with MS

**Figure 4:**
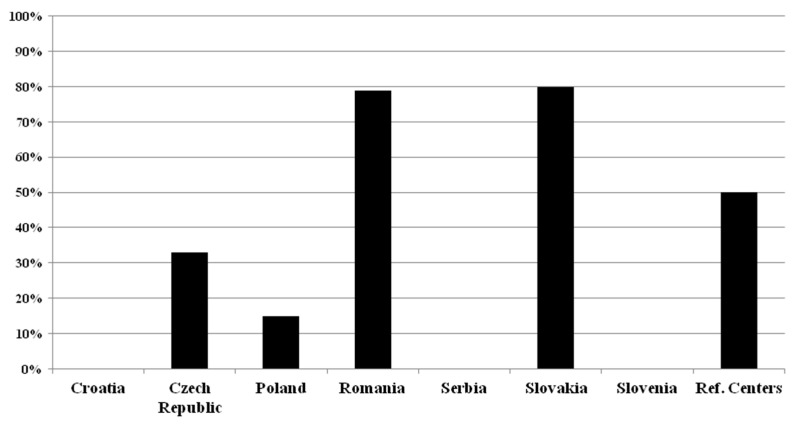
Neurofilament light chain (NfL) used as biomarker in MS in different countries.

None of the surveyed Croatian, Serbian, and Slovenian centers use neurofilament light chain or any other specific CSF and/or blood biomarkers in patients with MS (see Figure 4).

In Croatia; in Czech Republic; in Poland; in Romania; in Serbia; in Slovakia; in Slovenia; in the reference centers.

In the Czech Republic, only (3/9) 33% of the respondents use NfL for MS, and no others do. In Poland (3/20) 15% ask for the determination of NfL, and another one (1/20) (5%) uses Ig anti-AQP 4 and Ig anti-MOG in atypical cases, differentiation with NMOSD and MOGAD. In Romania, (11/14) 78.5% of respondents use NfL, and they also use IL17, miRNA, OCB, and IgG index. In Slovakia (4/5) 80% of the participating centers utilize NfL, and one (1/5, 20%) the HLA-DQB1 and HLA-DRB1. Half of the reference centers (2/4, 50%) request NfL; in addition, one center also uses the phenotyping of the CSF cells, transcriptomics, cytokines, and GFAP in the CSF. Most centers—which use NfL—request its determination from serum or sometimes from serum and CSF in parallel (Czech Republic: (2/3) 67% (from serum only) and (1/3) 33% (from both); Romania: (8/11) 73% and (3/11) 27%; Slovakia: (4/4) 100% and (0/4) 0%; reference centers: (1/2) 50% and (1/2) 50%), but two Polish centers (2/3, 67%) request it from CSF alone. The utilization rate of raw NfL concentrations versus age- and body mass index-corrected NfL Z scores was 3:1 in the Czech Republic, 2:1 in Poland, 4:6 in Romania; 4:0 in Slovakia; and 1:1 in reference centers. The purposes of the use of NfL are mostly the prognosis and monitoring of the disease course and evaluation of treatment response in Czech, Polish, and Romanian centers, while Slovakian centers utilize it for monitoring disease course and evaluation of treatment response. Reference centers use it only for monitoring disease course and for research purposes (see the details in Table 6).

Anti-drug antibodies (ADA) are determined regularly only by a smaller proportion of the centers; in contrast, the majority of the reference centers (3/4) (75%) use it. In Croatia, the ADA determination against Interferon-beta and Natalizumab is asked by only one center (1/5) (20%). The 44% (4/9) of the responding Czech centers request it; Natalizumab is asked for in the majority (4/4, 100%). In Poland—based on the received information—no center orders the ADA test against Interferon-beta, but in all centers that use Natalizumab, ADA against Natalizumab are determined regularly. In Romania, three centers (3/14, 21%) request, in which the Natalizumab determination is in higher proportion (Natalizumab/Interferon-beta 3:1). None of the surveyed Serbian centers ask for ADA determination. In Slovakia, the ADA determination against Interferon-beta is asked only by one center (1/5, 20%). None of the surveyed centers ask for its determination regularly in Slovenia. In contrast, the majority of the reference centers (3/4) (75%) use it, in equal proportion against Interferon-beta and Natalizumab.

## 3. Discussion

In the first part of the survey, we examined the organizational and institutional structures of the participating centers. Our study mostly involved larger Multiple Sclerosis Centers. CSF examination is not mandatory for diagnosing MS (it can be omitted in typical cases of CIS supported by characteristic MRI signs that clearly demonstrate dissemination in space and time, and the absence of atypical clinical and radiological features [4]). However, CSF analysis can aid in the diagnostic process [44]. According to the McDonald criteria, CSF examination is considered a “valuable diagnostic test”, mainly when clinical findings and MRI do not give enough proof for the diagnosis of MS, or in low-prevalence population or in case of primary progressive MS [4,45]. However, the centers we investigated considered it important as all surveyed centers of the participating countries always perform lumbar puncture for CSF analysis in patients with suspected MS, except the Romanian and Polish centers, where the opinions are somewhat divided. The centers primarily use it for both diagnostic and differential purposes. The routine CSF parameters usually requested by the surveyed centers in case of suspicion of MS are white blood cell count, red blood cell count, total protein, OCB, and intrathecal IgG. A short summary about routine CSF parameters can be found in the review of Deisenhammer et al. [46]. White blood cell count may indicate inflammation, while total protein or the albumin quotient can suggest blood–brain barrier dysfunction. CSF glucose concentration is normal in MS [47]. Lactate level in the CSF was found to be significantly elevated in MS patients compared to the control or to radiologically isolated syndrome [48,49], and it was positively correlated with the progression rate [50]. Intrathecally produced IgM and IgA inversely correlated with the disease progression in primary progressive SM [50].

Our responder centers use the CSF OCB determination. In the 2017 version of McDonald criteria the presence of IgG oligoclonal bands in CSF can perform the requirement of dissemination in time, so it makes for an early MS diagnosis in patients who fulfill the criteria for dissemination in space [1,4].

In our survey the opinions are quite divided on “What is the number of CSF-OCB that is used by their laboratory to define OCB positivity”. Only in Czech Republic 100% of the participants marked ≥2 CSF bands as the cut-off of OCB positivity. The lack of evidence-based recommendations in CSF OCB detection makes its analysis process and interpretation varied [51]. In the Higgins et al. [51] survey, the majority of Canadian neurologists preferred a cut-off of ≥2 CSF-specific bands as positive in agreement with the 2017 McDonald criteria [4].

To confirm intrathecal IgG synthesis, blood and CSF samples have to be analyzed in parallel. Quantitative IgG in CSF can be calculated by different formulae, such as IgG index [9,52,53], Reibergram [54,55], or Auer and Hegen formula [56]. In the calculation of intrathecal fraction most centers used the IgG index, but several also indicated Reibergram or other (Auer and Hegen formula).

Albumin quotient is a result of CSF albumin/serum albumin, and it characterizes the blood–brain barrier functions. IgG index, Reiber, and Auer and Hegen formulae include the determination of the Albumin quotient.

The IgG index value > 0.7 seemed to be an adequate cut-off value of the increased IgG index for MS patient population [14]. Its sensitivity was found to be lower than those of OCB [9,14].

The determination of κFLC in suspected MS cases by more than 50% of the centers occurs only in Czech Republic, Slovakia, Slovenia, and in the reference centers, and κFLC index is used by the majority in contrast to the absolute CSF κFLC concentration, except Romania and Slovakia. Previous studies have shown that both the absolute concentration of CSF-Kappa and the kappa index were found to have good MS diagnostic and prognostic performances [57,58,59,60,61]. κFree Light Chains are measured by turbidimetry or nephelometry and it is an easy, trusted, and fast tool, but cut-off values have to be determined [17,18].

Neurofilaments are neuron-specific cytoskeletal proteins and can be measured in both CSF and serum [23]. Our results showed that NfL is frequently used as a molecular biomarker for MS in Romania, Slovakia, and the reference centers. None of the surveyed Croatian, Serbian, and Slovenian centers used neurofilament light chain or any other specific CSF and/or blood biomarkers in patients with MS. Centers that use the NfL assay either measure serum only (dominantly), or both CSF and serum. They apply both NfL concentration or age- and body mass index-corrected Z scores in different proportions. Previous research showed that NfL was higher both in CSF and blood in newly diagnosed MS patients and its concentrations correlated with the disease activity, prognosis, and severity of the disease [9]. Higher NfL values are not specific to MS, as they can be found in other neurodegenerative disorders. It is also a possible biomarker to monitor treatment responses to DMTs [9]. The limitation is that the assays are not standardized, there are no accurate reference intervals or other factors defined for the result interpretation [9]. Different NfL cut-off values in clinical studies are summarized in the review of Kouchaki et al. [23]. NfL concentration as well as age- and body mass index-corrected Z scores are used, this latter takes into account the modifying effect of age and body mass index.

Based on the literature, we looked into the use of κFLC and NfL in some Western European countries. In France, κFLC index was found to be suitable in the diagnosis of MS [62,63,64] and it also proved to be an exact diagnostic marker in the pediatric MS population [65]. Additionally, it has been shown to be well used in predicting disease progression in CIS and RIS [66]. In contrast, based on a 6-year follow-up serum NfL and GFAP had only limited predictive value for MS outcomes [67]. In Italy κFLC (dominantly its index) was found to have high sensitivity in the diagnosis of MS [68,69]. This cost-effective method accomplished better than CSF IgG OCB in the diagnosis of MS/CIS [10,61,70]. Agnello et al. [71] proposed using the κFLC index as a “screening test” in suspected MS with using OCB serving as a “confirmatory test”. Higher plasma NfL values were associated with disease progression, while lower values were observed in patients treated with DMTs [72]. In Spain researchers [73,74,75] have recommended determining κFLC levels followed by OCB analysis as an additional test. The use of κFLC as a screening tool may reduce the number of manual OCB tests [76]. Higher serum NfL values were found to assist in the recognition of patients with higher risk of MS progression [77]. Furthermore, after 2 years use of Alemtuzumab treatment serum NfL levels were found to return to normal ranges [78].

Interferon-β and Natalizumab treatment may cause the production of antibodies. These interact with them and neutralize the aforementioned drugs, thus reducing their clinical and radiological effects [79,80]. In our survey, we found that anti-drug antibodies (ADA) were determined regularly only by a smaller proportion of the centers; in contrast, the majority of reference centers used it, both against Interferon-β and Natalizumab. Some European countries (but not all) used the ADA testing in clinical practice [81]. A survey about the use of antibodies against Interferon-beta and Natalizumab in different countries (Sweden, Austria, Denmark, Germany, Switzerland, and Spain) can be found in the paper of Link et al. [81]. Elevated baseline anti-Natalizumab antibody titers may predict reduced drug efficacy [82]. Monitoring anti-drug antibodies against DMTs can help in the prediction of later treatment failure [83].

Our results show that the use of molecular biomarkers is currently not uniform between the examined centers and countries. However, it can be seen that the role of molecular biomarkers has recently increased in the diagnosis and follow-up of MS. Further studies are needed to establish their possible role in routine clinical practice [2]. In the future, with the rise in personalized medicine, biomarker packages will presumably be included in the clinical diagnosis and follow-up of MS along with MRI and other clinical patient data.

The limitation of the present study is that we have not asked the reasons why a given diagnostic marker was not used. The study has not addressed the funding of laboratory biomarker tests in each country.

## 4. Materials and Methods

### 4.1. Participating Centers and Data Collection

Under the Danube Neurology Symposium for Neurological Sciences and Continuing Education, a Symposium for Multiple Sclerosis (SMS) was formed. The first part was to organize a survey about MS Registry. The Hungarian data have been published [84]. The publication about the international data are also available [85]. This international study was coordinated by the Department of Neurology, Albert Szent-Györgyi Faculty of Medicine, Albert Szent-Györgyi Health Centre, University of Szeged, Hungary and by the International Danube Neurology Symposium for Neurological Sciences and Continuing Education. Detailed information about the management and the organization of Multiple Sclerosis Centers in different countries can be found in our former publication’s Table 2 [85].

A questionnaire (edited by Professor Thomas Berger and Dr. Harald Hegen), consisting of 17 questions on CSF analysis and molecular biomarkers in MS patients, was sent via email to essentially the same MS centers that participated in our previous study [85] in the following countries: Croatia, Czech Republic, Poland, Romania, Serbia, Slovakia, and Slovenia. At the top of each questionnaire the respondents were required to provide their name and the name of their institution (see Acknowledgements section for a list of participating individuals and their institutions). Participation rates were as follows (number of the participating centers/whole number of the MS centers; participation rate): Croatia (5/10; 50%), Czech Republic (9/15; 60%), Poland (20/129; 15.5%), Romania (14/15; 93.3%), Serbia (5/5; 100%), Slovakia (5/10; 50%), and Slovenia (2/3; 66.6%). In addition, two reference centers from Austria (Department of Neurology, Medical University of Vienna and Department of Neurology, Medical University of Innsbruck), one center from Denmark (Danish Multiple Sclerosis Center, University Hospital Copenhagen, Rigshospitalet) and one from Germany (Department of Neurology, School of Medicine and Health, Technical University Munich) also completed our questionnaire; together, these form the reference center group. The MS prevalences/100,000 inhabitants are summarized in our former publication’s Table 4 by participating countries [85]. Our study mostly surveyed larger MS centers, which typically treat 30–100 and more than 100 patients per month. Data collection occurred between February 2024 and January 2025. The survey was conducted in the Hungarian centers, and the results are under submission.

### 4.2. Data Statistical Analysis

Descriptive statistics were used to analyze the data. Results are reported as the number of positively responding centers over the total number of responding centers in each country, expressed as percentages.

### 4.3. Ethical Approval

The study was approved by the Hungarian Medical Research Council (reference number: IV/5139-1/2021/EKU) in accordance with the Declaration of Helsinki.

## 5. Conclusions

Our study highlights the importance of CSF analysis in Multiple Sclerosis. Our surveyed centers request the determination of oligoclonal IgG bands in case of suspected MS, but there is no clear consensus in the number of CSF-restricted oligoclonal bands positivity within and between countries. In Czech Republic, in Slovakia, in Slovenia, and in the reference centers the determination of κFLC in patients with suspected MS is required by more than half of the surveyed centers. In Croatia, in Serbia, and in Slovenia none of the interviewed centers use NfL as biomarker in MS; in contrast, in Romania, in Slovakia, and in the reference centers it is often used. In summary, besides the use of CSF-specific oligoclonal bands there is no consensus among countries regarding the use of molecular biomarkers in Multiple Sclerosis, although neurologists find them useful. It is more likely a consequence of unavailability due to reimbursement issues. The role of molecular biomarkers is significant for diagnosis, progression assessment, and the effectiveness of therapy. Early diagnosis and prompt initiation of highly effective disease-modifying therapies offer the opportunity to delay long-term progression and preserve quality of life. In the future, biomarker-analyzing methods and reference (cut-off) values should be standardized, and a clear, uniform statement on their use would be necessary. It will be worthwhile to repeat the survey after about 3 years.

## Figures and Tables

**Table 1:** Various molecular biomarkers with current and potential clinical use in patients with suspected MS.

**Biomarkers used in clinical practice**
IgG OCB [1,3,6] Kappa free light chain (κFLC) [1,3,6] Neurofilament (mainly neurofilament light chain(NfL)) (it is not yet in McDonald criteria (2017)) [1,3,5,6]
**Potential biomarkers in MS**
Tau protein [1,3] Glial fibrillary acidic protein (GFAP) [1,3,6] S100β [1,3] Myelin basic protein (MBP) [1,3] Chitinase-3-like-1 (CHI3L1) [1,3,6] Chitinase-1 (CHIT1) [6] Osteopontin (OPN) [1,3,5] Matrix metallopeptidase-9 (MMP-9) [5] Soluble form of myeloid cells 2 (sTREM2) [6] Chemokine ligand (CXCL9, CXCL12, CXCL13) [1,3,5,6] CD163 [1] CD5+ B cells [1] Tubulin β [1] Heat shock protein 70, 90 (HSP70, HSP90) [1,3] Oncostatin M (OSM), Hepatocyte growth factor (HGS) [5] Neuron-specific enolase (NSE) [3] Cytokines (IL-6, IL-15) [3] Other lyphocyte subpopulation [3] Autoantibodies [3] Several microRNAs [3,6] Differentially expressed genes or proteins [3]

**Table 2:** Organization of the surveyed institutions presented by countries.

	*Neurology in-Bed* *Patients*	*A Specialized MS Clinic*	*A Research Agenda for MS and Biomarker Development*
***CROATIA***	**(5/5)100%**	**(3/5) 60%**	(2/5) 40%
***CZECH REPUBLIC***	**(8/9) 89%**	**(7/9) 78%**	(3/9) 33%
***POLAND***	**(18/20) 90%**	**(14/20) 70%**	(5/20) 25%
***ROMANIA***	**(14/14) 100%**	**(8/14) 57%**	(4/14) 29%
***SERBIA***	**(5/5) 100%**	**(5/5) 100%**	(0/5) 0%
***SLOVAKIA***	**(5/5) 100%**	**(4/5) 80%**	**(3/5) 60%**
***SLOVENIA***	**(2/2)100%**	**(2/2) 100%**	**(1/2) 50%**
***REFERENCE C.***	**(4/4) 100%**	**(4/4) 100%**	**(4/4) 100%**

Percentages ≥ 50% are marked with bold.

**Table 3:** CSF routine parameters usually performed in patients with suspected MS in different countries.

	*White Blood Cell Count*	*Red Blood Cell Count*	*Total* *Protein*	*OCB*	*Intrath IgG*	*Intrath* *IgA*	*Intrath* *IgM*	*CSF/se Glucose Ratio*	*CSF* *Lactate*
***CROATIA***	**(4/5)** **80%**	**(3/5)** **60%**	**(5/5)** **100%**	**(5/5)** **100%**	**(5/5)** **100%**	(0/5) 0%	(1/5) 20%	(1/5) 20%	(1/5) 20%
***CZECH*** ***REPUBLIC***	**(8/9)** **89%**	**(7/9)** **78%**	**(8/9)** **89%**	**(9/9)** **100%**	**(9/9)** **100%**	**(6/9)** **67%**	**(8/9)** **89%**	**(5/9)** **56%**	**(5/9)** **56%**
***POLAND***	**(17/20) 85%**	**(13/20) 65%**	**(19/20) 95%**	**(20/20) 100%**	**(19/20)** **95%**	(4/20) 20%	(8/20) 40%	**(11/20)** **55%**	(4/20) 20%
***ROMANIA***	**(12/14) 86%**	**(10/14) 71%**	**(12/14) 86%**	**(14/14) 100%**	**(12/14)** **86%**	(1/14) 7%	(2/14) 14%	**(9/14)** **64%**	(2/14) 14%
***SERBIA***	**(5/5)** **100%**	**(4/5)** **80%**	**(5/5)** **100%**	**(5/5)** **100%**	**(4/5)** **80%**	(0/5) 0%	(0/5) 0%	**(4/5)** **80%**	(1/5) 20%
***SLOVAKIA***	**(5/5)** **100%**	**(4/5)** **80%**	**(5/5)** **100%**	**(5/5)** **100%**	**(5/5)** **100%**	(2/5) 40%	**(3/5)** **60%**	(2/5) 40%	**(3/5)** **60%**
***SLOVENIA***	**(2/2)** **100%**	**(2/2)** **100%**	**(2/2)** **100%**	**(2/2)** **100%**	**(2/2)** **100%**	**(2/2)** **100%**	**(2/2)** **100%**	**(2/2)** **100%**	**(1/2)** **50%**
***REFERENCE*** ***CENTERS***	**(4/4)** **100%**	**(4/4)** **100%**	**(3/4)** **75%**	**(4/4)** **100%**	**(4/4)** **100%**	**(3/4)** **75%**	**(3/4)** **75%**	**(2/4)** **50%**	**(2/4)** **50%**

White blood cell count; Red blood cell count; Total protein; OCB: Oligoclonal bands; Intrathecal IgG synthesis; Intrathecal IgA synthesis; Intrathecal IgM synthesis; CSF/serum glucose ratio; CSF lactate. Percentages ≥ 50% are marked with bold.

**Table 4:** Parameters are considered as MS specific in different countries.

	*CSF-Restricted OCB*	*Intrathecal Kappa FLC*	*CSF Lymphocytosis*	*Other*	*None*
***CROATIA***	**(5/5) 100%**	(2/5) 40%	(0/5) 0%	(0/5) 0%	(0/5) 0%
***CZECH REPUBLIC***	**(9/9) 100%**	**(9/9) 100%**	(1/9) 11%	(0/9) 0%	(0/9) 0%
***POLAND***	**(17/20) 85%**	(3/20) 15%	(1/20) 5%	(1/20) 5%	(2/20) 10%
***ROMANIA***	**(8/14) 57%**	(4/14) 28.5%	(0/14) 0%	(0/14) 0%	(6/14) 43%
***SERBIA***	**(4/5) 80%**	(1/5) 20%	(0/5) 0%	(0/5) 0%	(1/5) 20%
***SLOVAKIA***	**(3/5) 60%**	(1/5) 20%	(1/5) 20%	(1/5) 20%	(2/5) 40%
***SLOVENIA***	**(1/2) 50%**	**(1/2) 50%**	(0/2) 0%	(0/2) 0%	**(1/2) 50%**
***REFERENCE C.***	**(2/4) 50%**	(1/4) 25%	(0/4) 0%	(0/4) 0%	**(2/4) 50%**

Percentages ≥ 50% are marked with bold.

**Table 5:** Number of CSF-restricted oligoclonal bands (OCB) used by laboratories to define OCB positivity in different countries.

	*≥1 CSF BANDS*	*≥2 CSF BANDS*	*≥3 CSF BANDS*	*≥4 CSF BANDS*	*OTHER ^#^*
***CROATIA***	(1/5) 20%	(2/5) 40%	(1/5) 20%	(0/5) 0%	(1/5) 20%
***CZECH REPUBLIC***	(0/9) 0%	**(9/9) 100%**	(0/9) 0%	(0/9) 0%	(0/9) 0%
POLAND *	(2/20) 10%	**(12/20) 60%**	(5/20) 25%	(1/20) 5%	(1/20) 5%
***ROMANIA***	(2/14) 14%	(4/14) 28%	(1/14) 7%	(1/14) 7%	(6/14) 43%
***SERBIA***	(0/5) 0%	**(3/5) 60%**	(2/5) 40%	(0/5) 0%	(0/5) 0%
***SLOVAKIA***	(1/5) 20%	**(4/5) 80%**	(0/5) 0%	(0/5) 0%	(0/5) 0%
***SLOVENIA***	**(1/2) 50%**	(0/2) 0%	(0/2) 0%	**(1/2) 50%**	(0/2) 0%
***REFERENCE C.***	(0/4) 0%	(1/4) 25%	(3/4) 75%	(0/4) 0%	(0/4) 0%

* 1 center marked both *≥2 CSF and ≥3 CSF bands*. ^#^ quantitative/semiquantitative analysis, only positive/negative. Percentages ≥ 50% are marked with bold.

**Table 6:** The purpose of NfL use in different countries.

	*Czech Republic* *3 = 100%*	*Poland* *3 = 100%*	*Romania* *11 = 100%*	*Slovakia* *4 = 100%*	*Reference Centers* *2 = 100%*
***Diagnosis of MS***	(0/3) 0%	(0/3) 0%	(1/11) 9%	(0/4) 0%	(0/2) 0%
***Prognosis of disease course***	**(3/3) 100%**	**(3/3) 100%**	**(8/11) 73%**	(0/4) 0%	(0/2) 0%
***Monitoring disease course***	**(3/3) 100%**	**(2/3) 67%**	**(11/11) 100%**	**(3/4) 75%**	**(1/2) 50%**
***Evaluation of treatment response***	**(2/3) 67%**	**(2/3) 67%**	**(11/11) 100%**	**(3/4) 75%**	(0/2) 0%
***As additional information***	(0/3) 0%	(1/3) 33%	(1/11) 9%	(1/4) 25%	(0/2) 0%
***Other***	(0/3) 0%	(0/3) 0%	(1/11) 9%	(1/4) 25%	**(1/2) 50%**

We considered 100% the number of the centers using NfL. Percentages ≥ 50% are marked with bold.

## Data Availability

The data that support the findings of this study are available from the corresponding author upon reasonable request.

## Abbreviations

The following abbreviations are used in this manuscript:

ADA	Anti-drug antibodies
CIS	Clinically isolated syndrome
CNS	Central nervous system
CSF	Cerebrospinal fluid
DMT	Disease-modifying therapy
Ig	Immunglobulin
κFLC	Kappa free light chain
LP	Lumbar puncture
MRI	Magnetic resonance imaging
MS	Multiple Sclerosis
NfL	Neurofilament light chain
OCB	Oligoclonal band
